# Perceived stigma among non-professional caregivers of people with severe mental illness, Bahir Dar, northwest Ethiopia

**DOI:** 10.1186/s12991-018-0212-4

**Published:** 2018-10-08

**Authors:** Temesgen Ergetie, Zegeye Yohanes, Biksegn Asrat, Wubit Demeke, Andargie Abate, Minale Tareke

**Affiliations:** 1 Department of Psychiatry, College of Medicine and Health Sciences, Bahir Dar University, Po box 79, Bahir Dar, Ethiopia; 2 Amanuel Specialized Mental Hospital, Addis Ababa, Ethiopia; 3 Department of Psychiatry, College of Medicine and Health Sciences, Gondar University, Gondar, Ethiopia; 4 College of Medicine and Health Sciences, Bahir Dar University, Po box 79, Bahir Dar, Ethiopia

**Keywords:** Caregivers, Perceived stigma, Severe mental illness

## Abstract

**Background:**

The stigmatization of mental illness is currently considered to be one of the most important issues facing caregivers of severely mentally ill individuals. There is a dearth of information about the prevalence and associated factors of perceived stigma among caregivers of people with severe mental illness in the study area.

**Objective:**

To assess the prevalence and associated factors of perceived stigma among non-professional caregivers of people with severe mental illness, Bahir Dar, northwest Ethiopia.

**Method:**

Institutional based cross-sectional study was conducted from May to June, 2016 at Felege Hiwot Referral Hospital among 495 caregivers of people with the severe mental illness. Pre-tested structured family interview schedule questionnaire was used. Binary logistic regression was applied to identify factors associated with perceived stigma and interpreted using odds ratio with 95% confidence interval. Statistical significance was considered at *p* value < 0.05.

**Result:**

The overall prevalence of perceived stigma was found to be 89.3%. Being female, rural residency, lack of social support, long duration of relationship with the patient and currently not married were found significantly associated with the perceived stigma of caregivers.

**Conclusion:**

Prevalence of perceived stigma is very high in the current study. Thus, stigma reduction program and expanding of strong social support should better be implemented by different stakeholders for caregivers of people with severe mental illness.

## Introduction

The burden of mental health problems is increasing globally [1]. Mental illness accounted for 13% of world’s disease burden and this figure will be increased to 15% by the year 2020 [2, 3]. Studies showed that approximately 450 million persons affected by mental illness and their devastating effects at personal and national levels are quite significant [1, 2, 4]. Due to different reasons, in low- and middle-income countries, about third quarter of people who need mental health service do not get any kind of intervention [5]. Stigma is one of the barriers that can prevent patients with mental illnesses from getting appropriate treatment or care [6].

Stigma is a social process, practiced or expected and characterized by separation, rejection, and blame or discredit about an individual or groups [7]. Stigma occurs at three levels, namely, organizational, public and personal level. Organizational stigma refers to the stigma that exists at system level which is defined as the rules, policies, and procedures of private and governmental entities in positions of power that restrict the rights and chances of people with disabling conditions [8]. Public stigma occurs at the group level and can be defined as the trends of massive social groups endorsing stereotype behavior and acting against a stigmatized group [9]. Personal level stigma is the stigma existing at the individual level. Perceived stigma existing at the personal level which is respondent’s beliefs in which those people with mental illness are generally stigmatized [10].

Facing stigmatization of mentally ill individual is one of the most important issues in mental health field until now [11]. It involves an isolation of person labeled as different from “us” who are believed to possess negative traits, resulting in negative feelings, discrimination, and status loss for the marginalized persons [12].

Feeling of stigmatization was not the only problem of people with severe mental illness, but also their family members who help care for them report feeling stigmatized as a result of their relationship with the loved one with mental illness [13–16]. Practically, 43–92% of the care providers of people with mental illness reported the feeling of being stigmatized [17]. In the United States, a study revealed that 43% of caregivers of people with mental illness perceived that they were stigmatized by others because of having a mentally ill individual within their relatives [15]. In Morocco, caregivers of schizophrenia patients reported high level of perceived stigma and faced serious impacts on their family members [18]. A community-based study conducted in Ethiopia showed that perceived stigma was 75% among caregivers of people with severe mental illness (SMI) [19]. Perceived stigma has affected caregivers of people with SMI in several ways including emotional, relationship, financial, health, and time stressors. They also described the feeling of being separated, ignored, blamed and criticized by peers, neighbors, coworkers and even mental health professionals [20]. Caregivers of people with mental illness are exposed to shame, low self-worth, and social isolation as a result of perceived stigma. Caregivers’ expectation of devaluation and discrimination from others leads them to adopt harmful coping mechanisms such as secrecy or withdrawal. As a result, caregivers hide patients, and patients may not get proper treatment or will be noncompliance [13].

A study revealed that perceived stigma of caregivers is associated with higher education, living with the patient in an urbanized area, being female, and a patient who had early age onset of illness, multiple admission and longer duration of the illness [14].

Even though studies around the globe demonstrated the high magnitude of perceived stigma, in Ethiopia, there are few previous studies that reported on perceived stigma specifically among non-professional caregivers of people with SMI.

Therefore, this study was proposed to determine the magnitude of perceived stigma and predictive factors among non-professional caregivers of people with SMI in the study area.

## Methods

### Study design and period

The facility-based cross-sectional study was conducted from May to June 2016 at Felege-Hiwot Referral Hospital (FHRH). The Hospital is found in the capital city of Amhara Regional state which is located 553 km far from the capital city of Ethiopia, Addis Ababa. It was established in 1963 as district hospital and changed into referral hospital in 2002. Psychiatry unit was established in 1990 and currently, there are five psychiatry outpatient units which provide services for about 70–90 patients per day.

### Sample size determination and sampling procedure

#### Sample size and Sampling procedures

The sample size was determined using single-population proportion formula $[n\, = \,{{\left( {\left( {z\alpha / 2} \right) 2p\left( { 1\, - \,p} \right)} \right)} \mathord{\left/ {\vphantom {{\left( {\left( {z\alpha / 2} \right) 2p\left( { 1\, - \,p} \right)} \right)} {d2}}} \right. \kern-0pt} {d2}}]$ with the following assumptions: 95% confidence interval (CI) (*Zα*/2 = 1.96) and the proportion (*p*) of perceived stigma of caregivers to be 75% from previous study at Buta Jira [19], marginal error 4%. By adding 10% non-response rate, a total of 495 study populations were involved.

The study participants were selected using systematic random sampling technique. The numbers of patients with SMI who had monthly regular follow-up estimated were 1482; of these, 526 people with schizophrenia, major depressive disorder (MDD) (600) and bipolar disorders (356). The total sample size was allocated proportionally to each SMI patient caregivers. Sampling fraction was 3 (i.e. 1482/495 ≈ 3) for all samples. Lottery method was used to get the first participant who arrived at outpatient settings for each type of severely mentally ill patients’ caregivers. Then after, ever 3rd caregivers were interviewed according to their arrival in outpatient settings for each type of caregivers independently. A caregiver who provides more care was interviewed when the patient had more than one caregiver.

### Operational definitions

#### Caregiver

Someone who provided more than 6 months of care and regularly responsible for taking care of patients more than other immediate or non-immediate family relative rather than by a health professional.

#### Immediate family relative

Father/mother, son/daughter and brother/sister.

#### Non-immediate family relative

Other relatives and friends.

#### Severe mental illness

Mental illness that includes schizophrenia, major depressive and bipolar disorders.

### Data-collection instruments and procedures

Data were collected using standardized Family Interview Schedule (FIS) questionnaire, which was developed as part of a world health organization study on the course and outcome of schizophrenia [21]. FIS questionnaire was 14-item questions regarding stigma that might affect families. Each stigma item was rated on a four-point scale, not at all (0), sometimes (1), often (2) and a lot (3) with respect to stigma. To assess the distribution of stigma responses between groups, a stigma sum score was computed by summarizing all positive responses (≥ 1) for each of the 14 items. The presence of just one positive answer on the stigma questionnaire was enough to represent a form of perceived stigma [19].

Social support was measured using the Oslo-3 Social Support Scale (OSS-3) with three questions. We used the sum score scale ranging from 3 to 14, which has three broad categories: “poor support” 3–8, “moderate support” 9–11 and “strong support” 12–14 [22].

A semi-structured questionnaire was used to collect socio-demographic, relationship and clinical factors. Data were collected using pre-tested structured questionnaires using face to face interview. Five data collectors (BSc nurses) and one MSc in mental health supervisor were recruited to conduct face to face interview of the caregivers for a month duration.

### Data quality management

Data quality control issue is ensured by conducting pre-test among 25 caregivers in the study area 1 week before the actual data-collection periods. One-day training was given for data collectors and supervisors on how to use the questionnaires, how to approach the participants of the study and about the purpose of the study. In addition to this, English questionnaires were translated to local language respondents’ Amharic language. Supervisor and principal investigator were closely followed the whole period of data-collection process. Supervision was held regularly during the data-collection period. Collected data were checked on a daily basis for its completeness.

### Data processing and analysis

Data were checked, coded, cleaned and entered into Epi-Info version 7 and exported to SPSS version 20 for analysis. Frequency, percentage, mean, standard deviation, tables, and charts were used to report the results of the data. Bivariate logistic regression analysis used to examine the association between dependent and independent variables. All variables with *p* < 0.2 in bivariate analysis were fitted into the multivariate logistic regression model to identify factors associated with perceived stigma. The association was interpreted using odds ratio and 95% confidence interval. *p* < 0.05 was considered statistically significant in this study.

### Ethical consideration

Ethical clearance obtained from University of Gondar ethical review board committee before data-collection period. The official letter was obtained and given for Amhara regional health bureau and Felege-Hiwot Referral Hospital. The study participants were informed about the purpose of the study. Written informed consent was obtained from participants during data-collection period, and they were informed that participation was on the voluntary basis and had full right to withdraw at any time during the interview process. They were also informed that refusal to participate had no negative consequences on the patients’ care, and participation had no financial benefit. Confidentiality was maintained throughout the study.

## Results

### Socio-demographic characteristics

Out of 495 recruited caregivers, 478 participated in the study yielding a response rate of 96.56%. The mean age of caregivers was 37.08 (± 13.4 SD) years. Nearly half of caregivers were females 244 (51%) and rural residents 262 (54.8%). Majority of respondents were Amhara by ethnicity 469 (98.1%), Orthodox religion follower 384 (80.3%) and currently not married 374 (78.2%) (Table 1).

**Table 1:** Socio-demographic characteristics of caregivers at Felege-Hiwot Referral Hospital, Bahir Dar, northwest Ethiopia, July, 2016 (*n* = 478)

Variables	Categories	Frequency	Percent
Age	18–24	89	18.6
	25–34	138	28.9
	35–44	108	22.6
	44–54	81	16.9
	≥ 55	62	13
Sex	Male	234	49
	Female	244	51
Residence	Urban	216	45.2
	Rural	262	54.8
Ethnicity	Amhara	469	98.1
	Others^a^	9	1.9
Religion	Orthodox	384	80.3
	Muslim	88	18.4
	Others^b^	6	1.3
Marital status	Currently not married	374	78.2
	Currently married	104	21.8
Educational status	Unable to read and write	121	25.3
	Primary	115	24.1
	Secondary	90	18.8
	Diploma and above	152	31.8
Job	Government employee	73	15.3
	Private employee	72	15.1
	Merchant	65	13.6
	Farmer	143	29.9
	House wife	71	14.9
	Student	54	11.3

Others^a^ = Tigrie, Oromo and Guragie; Others^b^ = Protestant and Catholic

### Psychosocial factors

Nearly half of the caregivers had poor social support 260 (54.4%), others had moderate social supports 136 (28.5%), and strong social supports 82 (17.1%).

### Relationship factors

Caregivers who have the duration of relationship with the patient for about 20–39 years were 238 (49.8%) and not live together with the patient 279 (58.4%) (Table 2).

**Table 2:** Relationship factors of caregivers of people with SMI at Felege-Hiwot Referral Hospital, Bahir Dar, northwest Ethiopia, July, 2016 (*n* = 478)

Variables	Categories	Frequency	Percent
Type of relationship	Mother	64	13.4
	Father	113	23.6
	Spouse	98	20.5
	Child	75	15.7
	Brother/sister	83	17.4
	Other relatives/friends	45	9.4
Duration of relationship with the patient (years)	0–19	181	37.9
	20–39	238	49.8
	40–59	59	12.3
Do you live together with patients	Yes	199	41.6
	No	279	58.4

### Clinical factors

Almost half of patient’s illness onset was below the ages of 20 years 262 (54.8%) and 307 (64.2%) had history of admission (Table 3).

**Table 3:** Clinical factors of caregivers of people with SMI at Felege-Hiwot Referral Hospital, Bahir Dar, northwest Ethiopia, July, 2016 (*n* = 478)

Variables	Categories	Frequency	Percent
Types of diagnosis	Schizophrenia	175	36.6
	Bipolar disorders	115	24.1
	MDD	188	39.3
Age of illness onset (years)	≤ 20	262	54.8
	21–40	135	28.2
	≥ 40	81	16.9
Duration patient’s Illness (years)	≤ 1	111	23.2
	> 1	367	76.8
Duration of treatment of patients (years)	≤ 1	205	42.9
	2–5	178	37.2
	6–10	75	15.7
	≥ 11	20	4.2
Admission	No	171	35.8
	Yes	307	64.2

### Prevalence of perceived stigma

The prevalence of perceived stigma in our study was 89.3% (95%, CI 84.6, 91.9). The mean and standard deviation of FIS scale was 7.6 and 8.1 respectively and its minimum and maximum value ranges from 0 to 34. Regarding the proportion of perceived stigma toward each item, three quarters (75.2%) of the caregivers agreed with the item “Felt grief or depression because of it”, followed by ‘‘Helping other people to understand what it is like to have a family member with psychiatric problem’ ‘(43.9%). The least frequently endorsed item was ‘felt that somehow it might be your fault’ (14.6%) (Table 4).

**Table 4:** Proportion of perceived stigma response of caregivers to each item at Felege-Hiwot Referral Hospital, Bahir Dar, northwest Ethiopia, July, 2016 (*n* = 478)

S. no	Items	Negative responses	Any positive response			
		Not at all total (%)	Some times	Often	A lot	Total (%)
1	Worried about being treated differently	365 (76.4%)	58	14	41	113 (23.6%)
2	Worried people would know about it	352 (73.7%)	46	27	53	126 (26.3%)
3	Felt the need to hide this fact	356 (74.4%)	52	21	49	122 (25.6%)
4	Helping other people to understand what it is like to have a family member with psychiatric problem	268 (56.1%)	134	33	43	210 (43.9%)
5	Making an effort to keep this fact a secret	349 (73%)	57	23	49	129 (27%)
6	Worried about being avoided	381 (80%)	46	24	27	97 (20%)
7	Explaining to others that (name) isn’t like their picture of “crazy” people	341 (71.3%)	85	25	27	137 (28.7%)
8	Worried that people would blame you for his or her problems	401 (83.9%)	44	18	15	77 (16.1%)
9	Worried that a person looking to marry would be reluctant to marry into your family	352 (73.6%)	54	20	52	126 (26.4%)
10	Worried about taking him or her out	359 (75.1%)	48	17	54	126 (24.9%)
11	Felt ashamed or embarrassed about it	335 (70.1%)	125	17	1	143 (29.9)
12	Sought out people who also have a family member who has had psychiatric problem	301 (63%)	79	20	78	177 (37%)
13	Felt grief or depression because of it	119 (24.9%)	140	61	158	359 (75.1%)
14	Felt that somehow it might be your fault	408 (85.4%)	50	12	8	70 (14.6%)

### Associated factors with perceived stigma

In the bivariate logistic regression analysis, sex, residence, social support, age of onset of illness, patient’s duration of treatment, marital status, care giver’s educational level, patient’s numbers of hospital admission and duration of relationship with the patient were significantly associated with previewed stigma at *p* value < 0.2 level and entered for further analysis into multivariate logistic regression to control confounding factors. On the other hand, ethnicity, religion, types of diagnosis, duration of illness, the age of respondents, job, types of the relationship of caregivers with the patient and living together or not living together were not statistically significant. In the logistic regression, after controlling confounding factors, sex, residency, social support, marital statuses, and numbers of admission and duration of relationship with the patient were found statistically significant.

Female caregivers were three times more likely to have perceived stigma compared to male caregivers (AOR = 3.02, 95% CI 1.30, 7.11), not currently married were three times more likely to have perceived stigma compared to currently married caregivers (AOR = 3.20, 95% CI 1.48, 6.91) and caregivers who lived in rural were three times more likely to have perceived stigma compared to who lived in urban areas (AOR = 2.80, 95% CI 1.20, 6.54). Caregivers who had poor social support were five times more likely to have perceived stigma compared to caregivers who have strong social support (AOR = 5.06, 95% CI 1.96, 13.13).

Caregivers who gave care for patients for 20–39-year duration were five times more likely to have perceived stigma compared to those who gave care for 6 months–19 years of duration (AOR = 4.92, 95% CI 1.30, 18.67). In addition to this, caregivers who cared for the patient who had the history of admission were 77% less likely to have perceived stigma compared those who gave no history of admission (AOR = 0.23, 95% CI = 0.10, 0.51) (Table 5).

**Table 5:** Factors associated with perceived stigma among caregivers of people with SMI attending at Felege-Hiwot Referral Hospital, Bahir Dar, northwest Ethiopia, July, 2016 (*n* = 478)

Explanatory variables	Category	Perceived stigma		COR (95% CI)	AOR (95% CI)
		Yes	No		
Sex	Male	199	35	1.0	1.0
	Female	228	16	2.51 (1.35, 4.67)	3.03 (1.29, 7.11)^a^
Residency	Urban	187	29	1.0	1.0
	Rural	240	22	1.70 (0.94, 3.304)	2.80 (1.20, 6.54)^a^
Social support	Poor	244	16	5.59 (2.77, 11.30)	5.06 (1.96, 13.13)^a^
	Moderate	123	13	3.47 (1.64, 7.36)	2.43 (0.91, 6.45)
	Strong	60	22	1.0	1.0
Age onset of illness (years)	≤ 20	254	8	1.0	1.0
	21–39	100	35	0.09 (0.04, 0.20)	0.47 (0.75, 8.12)
	≥ 40	73	8	0.29 (0.10, 0.79)	0.40 (0.14, 1.13)
Duration of treatment (years)	< 1	191	14	1.0	1.0
	2–5	159	19	0.61 (0.30, 1.26)	0.22 (0.64, 16.22)
	6–10	64	11	0.43 (0.18, 0.99)	0.14 (0.62, 15.85)
	≥ 11	13	7	0.14 (0.05, 0.40)	0.12 (0.39, 11.64)
Marital status	Currently not married	350	24	5.11 (2.80, 9.34)	3.20 (1.48, 6.91)^a^
	Currently married	77	27	1.0	1.0
Educational level	Unable to read and write	102	19	0.46 (0.21, 0.99)	1.00 (0.35, 2.86)
	Primary	106	9	1.01 (.41, 0.2.48)	1.45 (0.49, 4.26)
	Secondary	79	11	0.62 (0.26, 1.46)	0.74 (0.26, 2.06)
	Diploma and above	140	12	1.0	1.0
History of admission	No	146	25	1.0	1.0
	Yes	281	26	1.85 (1.03, 3.32)	0.23 (0.10, 0.51)^a^
Duration of care for the patient	6 moths–19 years	149	32	1.0	1.0
	20–39 years	227	11	4.43 (2.17, 9.10)	4.92 (1.30, 18.67)^a^
	≥ 40 years	51	8	1.37 (0.060, 3.16)	3.80 (1.20, 11.98)^a^

*COR* crude odds ratio, *AOR* adjusted odds ratio

^a^Statistically significant at *p* value < 0.05

## Discussion

The increment in the prevalence of perceived stigma globally needs a better understanding of the local burden and most common influencing factors. In the current study, the overall prevalence of perceived stigma among caregivers of people with SMI was found to be 89.3% which was in line with the study done in Morocco which was 86.7% [18].

However, the prevalence in this study was higher than the prevalence reported in the United States (43%), Belgium (86%) and Butajira, Ethiopia (75%) [15, 19, 23]. The difference might be due to variation in sample size, instruments they used, cultural, socio-demographic characteristics of participants and study population. In addition to this, perceived stigma in our study might be due to a misperception about mental illness and most of the time people believed that mental illness is happened as a result of supernatural punishment. Furthermore, the sample size in Butajira was 178 and conducted at the community level, however, the current study was done at health institution among 478 caregivers of people with SMI. There was also an educational status difference.

In this study, perceived stigma among female caregivers was higher than male caregivers, which was in line with the previous studies done in China and American [14, 24]. This might be due to the reason in which the role of caring and social burden for the female is more burdensome increasing their vulnerability to perceived stigma.

Caregivers who were currently not married and lived in the rural area were three times more likely to have perceived stigma compared to those who were currently married and lived in the urban area. This result has concurred with the findings documented in India and China [25, 26]. This might be due to lack of intimate social support to share stressful feelings, having low self-esteem and poor coping mechanism which are common among not married people exposing them to have perceived stigma. This thought is supported by the current study stating that poor social support increased the level of perceived stigma. In addition, caregivers who lived in the rural area experienced the high level of perceived stigma. These could be due to rural residents’ lack of awareness and cultural belief about the causes of mental illness such as spiritual possessions, the result of a sinful act or punishment from God [27].

Caregivers who had poor social support were five times more likely to have perceived stigma compared to those with strong social support. This result is in line with the data from a study conducted in China [26]. The possible reason could be the assumptions that caregivers who had a mentally ill person with family members might isolate themselves from the societies and cannot share different roles, responsibilities and their feelings.

Regarding duration of patient care, those who gave care for longer duration were more likely to have perceived stigma than those who gave care for a relatively shorter duration which is in line with the previous result in China [14]. Perception of stigma may be increased when the duration of caring increased. Because caring for people with severe mental illness causes a burden for caregivers in every aspect of life including economic, social, financial, physical and psychological consequences.

Admitted patient caregivers had a lower level of perceived stigma compared to caregivers who had no SMI family members with a history of admission. The current finding is inconsistent with the previous institution-based study was done in China [14]. The difference might be due to the difference in study design (follow-up study), measurement tool (Camberwell Family Interview) and study population (schizophrenia caregivers) in China. Moreover, the information might create awareness since they exchange information about mental illness with different health professionals and other peoples who cared people with several mental illnesses during their hospital stay.

### Limitation of the study

Since the study was conducted only using quantitative design, it might not explore well the perception of caregivers’ perceived stigma. In addition, a way of using an interpretation tool may exaggerate the presence of perceived stigma. Most comparison parts of discussions are not culturally matched.

## Conclusion

This study finding showed a high prevalence of perceived stigma among caregivers of people with severe mental illness at Felege-Hiwot Referral Hospital, Bahir Dar. Being female, rural residency, poor social support, currently not being married and long duration of caring for patient were found to be significantly associated with the perceived stigma of caregivers of people with the severe mental illness. Therefore, it is very important to increase strong social support towards caregivers of people with mental illness by collaborating with different stakeholders and link them to support groups such as nongovernmental organizations and social workers, and to formulate certain interventions that focus on reduction of perceived stigma among caregivers.
